# Evaluation of the living with hope program for rural women caregivers of persons with advanced cancer

**DOI:** 10.1186/1472-684X-12-36

**Published:** 2013-10-09

**Authors:** Wendy Duggleby, Allison Williams, Lorraine Holstlander, Dan Cooper, Sunita Ghosh, Lars K Hallstrom, Roanne Thomas McLean, Mary Hampton

**Affiliations:** 1 Nursing Research Chair Aging and Quality of Life, Faculty of Nursing University of Alberta, 3rd Level ECHA 11403 87th Ave, Edmonton, AB T6G 1C9, Canada; 2 CIHR ECHO/IGH Mid Career Scientist, School of Geography and Earth Sciences, McMaster University, 1280 Main Street, West Hamilton ON L8S 4K1, Canada; 3 College of Nursing, University of Saskatchewan, Office Rm 343 Ellis Hall 107 Wiggins Rd, Saskatoon, SK S7N5E5, Canada; 4 RQHR Palliative Care Services, Clinical Practice Leader (Spiritual Care), Spiritual Care Educator, Research, QI and Special Projects Manager, 4F - 4101 Dewdney Ave, Regina, SK S4T 1A5, Canada; 5 Medical Oncology, University of Alberta, Cross Cancer Institute, 11560 - University Ave NW, Edmonton, AB T6G 1Z2, Canada; 6 University of Alberta, Augustana Campus, Room 2-134 Augustana Forum, 4901-46 Ave, Camrose, AB T4V 2R3, Canada; 7 Chaire de recherche du Canada, Professeure agrégée, École des sciences de la réadaptation Université d'Ottawa, Guindon Hall, Room 3068, 451 Smyth Rd, Ottawa, ON K1H 8M5, Canada; 8 Psychology, Luther College, University of Regina, 3737 Wascana Parkway, Regina, SK S4S 0A2, Canada

**Keywords:** Hope, Caregivers, Palliative care, Intervention

## Abstract

**Background:**

Hope has been identified as a key psychosocial resource among family caregivers to manage and deal with the caregiver experience. The Living with Hope Program is a self-administered intervention that consists of watching an international award winning *Living with Hope* film and participating in a two week hope activity (“Stories of the Present”). The purpose of this study was to examine the effects of the Living with Hope Program on self-efficacy [General Self-Efficacy Scale], loss and grief [Non-Death Revised Grief Experience Inventory], hope [Herth Hope Index] and quality of life [Short-Form 12 version 2 (SF-12v2)] in rural women caring for persons with advanced cancer and to model potential mechanisms through which changes occurred.

**Methods:**

A time-series embedded mixed method design was used, with quantitative baseline outcome measures repeated at day 7, day 14, and 3, 6 and 12 months. Qualitative data from the hope activity informed the quantitative data. Thirty-six participants agreed to participate with 22 completing all data collection. General estimating equations were used to analyze the data.

**Results:**

Herth Hope Index scores (p=0.05) had increased significantly from baseline at day 7. General Self Efficacy Scale scores were significantly higher than baseline at all data time points. To determine the mechanisms of the Living with Hope Program through which changes occurred, results of the data analysis suggested that as General Self Efficacy Scale scores increased (p<0.001) and Non-death Revised Grief Experience Inventory scores decreased (p=0.01) Herth Hope Index scores increased. In addition as Herth Hope Index scores increased (p<0.001) and Non-death Revised Grief Experience Inventory scores decreased (p=0.01), SF-12v2 mental health summary scores increased. Qualitative data suggested that through the hope activity (Stories of the Present) the participants were able to find positives and hope in their experience.

**Conclusions:**

The Living with Hope Program has potential to increase hope and improve quality of life for rural women caregivers of persons with advanced cancer. The possible mechanisms by which changes in hope and quality of life occur are by decreasing loss and grief and increasing self-efficacy.

**Trial registrations:**

Registration ClinicalTrails.gov, NCT01081301.

## Background

Research studies have clearly established the negative consequences associated with caring for a family member at the end of life [1,2]. However, despite the critical need to support these caregivers, there is a paucity of research evaluating the effectiveness of supportive interventions [1]. Moreover, intervention studies have not focused on the most vulnerable of caregivers: women living in rural areas. Family caregivers, who do not have access to palliative services (including counselling and bereavement services), such as those in rural areas, are in need of more support than other populations [3]. As well caregiving has been found to have a greater impact on the health of women than on the health of men [4]. Hope has been identified as a key psychosocial resource among family caregivers to manage and deal with the caregiving experience [5,6]. It has been defined by caregivers as the inner strength to achieve future good and to continue care giving [6]. When the hope of family members and palliative care patients are compared, levels of hope were found to be significantly lower for family members than patients [7]. As well, patient and caregivers had different perspectives on hope [8]. Given these findings, interventions to foster hope that are specifically tailored to family caregivers of persons with advanced cancer are important for supporting this population.

Hope has a positive influence on family caregivers’ quality of life. As the hope of caregivers increases, so does their quality of life [9,10]. Correspondingly, hopelessness (low levels of hope) can reduce caregivers’ quality of life [11-15]. Supportive hope programs have been found to increase hope and quality of life in other populations [16,17]. Thus a psychosocial supportive hope fostering program may support and sustain women caring for family members with advanced cancer.

A Living with Hope Program for family caregivers was developed and pilot tested by the authors [18]. The Living with Hope Program is a self-administered intervention that consists of watching an international award winning *Living with Hope* film and taking part in a two week hope activity (“Stories of the Present”). Pilot test findings suggests the Living with Hope Program is an acceptable and feasible intervention that shows promise for increasing hope and quality of life in family caregivers of persons with advanced cancer. The purpose of this study was to further evaluate the Living with Hope Program in rural women caregivers of persons with advanced cancer.

### Conceptual model

**Figure 1:**
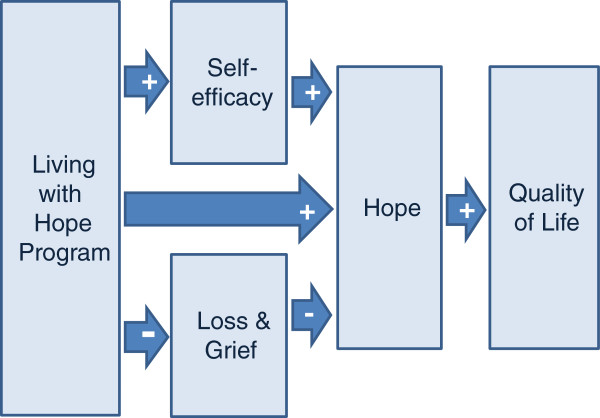
Living with Hope Conceptual Model.

The conceptual model for this study (Figure 1) incorporates Social Cognitive Theory [19] and the conceptual model entitled “Hanging on to Hope” [6]. “Hanging on to Hope” was developed through a grounded theory study of family caregivers of persons with advanced cancer. In this model, loss and grief resulted in loss of hope for family caregivers. Participants described their feelings of loss and grief for the physical changes their family member was experiencing and changes in their relationships. The basic social process of family caregivers of persons with advanced cancer was “writing their own story”. This process was described by the study participants as a way to maintain self-efficacy and increase their hope. Self-efficacy is defined as the confidence in the ability to deal with difficult situations [20].

Graves [21], in a meta-analysis of psychosocial intervention components, found interventions that focused on increasing self-efficacy (the belief in a persons’ ability to organize and execute actions) influenced a person’s psychological and physiological functioning (health status)^.^ We hypothesized that participants would report increased self-efficacy, decreased grief and loss and increased hope and quality of life, as compared to baseline, after participating in the Living with Hope Program. More specifically, we hypothesized that administration of the Living with Hope Program would improve self-efficacy and decrease feelings of loss and grief, leading to a positive influence on the proximal outcome of hope and the distal outcome of quality of life.

### Purpose

The purpose of this study was to examine the effects of the Living with Hope Program on self-efficacy [General Self-Efficacy Scale (GSES)], loss and grief [Non-Death Revised Grief Experience Inventory (NDGREI)], hope [Herth Hope Index (HHI)] and quality of life [Short-Form 12 version 2 (SF-12v2)] in rural women caring for persons with advanced cancer. The specific aims of the study were to:

1) Examine patterns of changes of the main variables compared to baseline over time (day 7, 14, 3, 6 and 12 months).

2) Determine the mechanisms of the Living with Hope Program by testing the study conceptual model (Figure 1), in which self-efficacy and loss and grief are hypothesized intermediary variables for changes in hope, and subsequently quality of life among rural women caring for persons with advanced cancer.

3) Describe the participants’ perceptions of what fosters their hope.

## Methods

**Figure 2:**
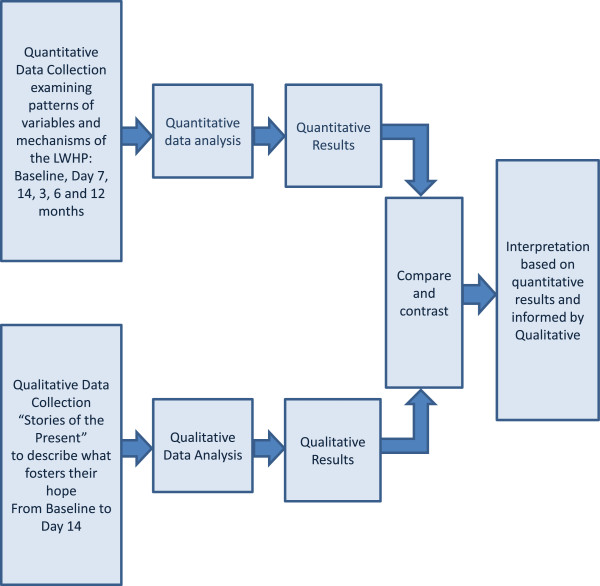
Study Design.

A time-series embedded mixed method design (Quant+qual) was used to achieve the study purpose and aims (Figure 2). In the embedded explanatory mixed method design, the qualitative data plays a supplementary role [22]. Each data set is analyzed separately and the findings integrated in the results. In this study, baseline outcome variables were measured quantitatively, followed by implementation of the intervention (Living with Hope Program) which was given to all participants. Participants were then followed over time with repeated measures of outcome variables. The qualitative data, embedded in the intervention, was collected as part of the Living with Hope Program in the form of a hope directed journaling activity entitled “Stories of the Present”. The study received ethical approval from Alberta Cancer Research Ethics Board, University of Saskatchewan Behavioral Ethics Review Board and the Regina Qu’Appelle Health Region Research Review Board.

### Living with hope program

The Living with Hope Program consisted of: a) viewing the Living with Hope film which features caregivers of patients with advanced cancer describing their hope and b) a hope activity entitled “Stories of the Present”. The hope activity involved participants writing about their challenges, what gave them hope and what they felt would give them hope. Participants were encouraged to write their “Stories of the Present” over a two week time period. The two week time period was based on a review of journaling studies and older adults which suggested that the optimum length of time for journaling is between one and two weeks [23]. The dosage (amount of the intervention received) of the Living with Hope Program was determined by the number of journal entries.

### Measures

#### Herth hope index (HHI)

This 12 item scale measuring hope provides a total summary score and three sub-scales scores: a) temporality and future, b) positive readiness and expectancy, and c) interconnectedness [24]. These three subscales are consistent with descriptions of hope by caregivers in the preliminary work completed by the research team. Summative scores range from 12–48, with a higher score denoting greater hope. Reliability (test-retest) is reported to be r=0. 91, p<0. 05 and validity (concurrent validity) at r=0. 84, p<.0 05; (criterion), r=. 92, p< 0.05; (divergent), r=−.73, p<0. 05) [5,24].

#### General self efficacy scale (GSES)

This scale consists of 10 items with responses from 0–4. Higher the scores on the General Self Efficacy Scale, which has a maximum score of 40, indicate higher participant feelings of self-efficacy. The General Self Efficacy Scale was chosen as a measure for this study because it has been found to be a reliable and valid measure in many populations [20]. It has been used successfully in a study of male caregivers of persons with breast cancer [25].

#### Short form 12 (SF-12v2) version 2

The SF-12v2 does not produce a total quality of life summary scores, but a physical component summary score (PCS), and a mental health summary score (MCS). The PCS and MCS have a maximum score of 100. These components of the SF-12v2 correlate very highly (0.95 and 0.96) with the SF-36 [26].

#### Non-death revised grief experience inventory (NDRGEI)

The Non-Death Revised Grief Experience Inventory measures grief that is not associated with the death of a person. It is a 22-item scale measuring four domains (existential concerns, depression, tension and guilt, and physical distress) of the grief experience. Responses are scored on a 6-point scale, ranging from slight disagreement to strong agreement, with higher the total score indicating more grief and loss. The Non-Death Revised Grief Experience Inventory has a maximum score of 132. The scale has established reliability (alpha =0. 93) and validity (p=0. 001) [27]. This scale was used in a study of hope and caregivers [5].

#### Data collection form

Data regarding the journals (approximate daily time spent journaling, number of journal entries), and possible co-interventions such as support groups were collected using this form.

### Sample and setting

Family caregivers in this study were defined broadly as a family member or significant other identified by the patient as his or her primary source of emotional and physical support. Rural was defined as living outside major population areas in Alberta and Saskatchewan with rural areas designated by provincial postal codes [28].

#### Inclusion and exclusion criteria

Sample inclusion criteria were: a) female, b) 18 years of age and older, c) caring for a family member who has a diagnosis of advanced cancer and has been referred to palliative care and /or is receiving palliative care services, d) home address is a rural postal code, and e) English speaking. Exclusion criteria were a) women who were cognitively impaired as determined by the recruitment team at the site, b) women otherwise unable to participate, in the opinion of the recruitment team and c) women caring for a family member who has a diagnosis of advanced cancer as well as dementia.

#### Sample size

Sample size was determined based on a power of 0.80, alpha of 0.05, and a moderate effect size of 0.5. Using Munro’s [29] tables for power analysis an adequate sample size would be 48. Convenience sampling was used. Thirty-six participants consented to participate. The sample was recruited using multiple strategies. Potential participants were mailed invitations to participate by the Saskatchewan and Alberta Cancer Registries. If they returned their contact information in a prepaid postage envelope, they were contacted by a research assistant (RA) to discuss the study. In Saskatchewan, the Palliative Care Admission team in Regina Qu’Appelle Health Region and nurses at the Saskatchewan Cancer Agency also identified potential participants. In Alberta, the Alberta Health Services Cancer Care and Community Cancer Clinics in rural communities also identified potential participants. Those who agreed were contacted by trained RAs (experienced Registered Nurses) to arrange a time to meet to discuss the study.

### Data collection

The study protocol has been published previously [30]. At the first visit (baseline) a written informed consent was obtained from the person with advanced cancer and their family caregiver. Demographic information of the caregiver and family member was then collected followed by baseline measures of hope, quality of life, self-efficacy and loss and grief. All subjects received the Living with Hope Program. At day 7 and 14, and 3, 6 and 12 months, data were collected as per baseline. Participants were also asked additional questions such as how much time they spent during the week on their hope activity. At Day 14 “Stories of the Present” were photocopied with the permission of the participants. Trained Registered Nurses (inter rater reliability 100%) collected data at baseline, day 7 and day 14 in the participant’s homes. Data were collected at 3, 6 and 12 months via telephone.

### Analysis

Qualitative data (Stories of the Present) were transcribed by an experienced transcriptionist and entered into NVivo for data management. All quantitative data were cleaned and checked and entered into SPSS V19. SF-12v2 summary scores were calculated using Quality Metric software [31].

#### Specific aim #1

Generalized estimating equations were used to determine change in patterns of General Self Efficacy Scale, Non Death Revised Grief Experience Inventory, Herth Hope Index and SF-12v2 Physical and Mental health scores over time (Day 7, 14 and 3, 6, and 12 months) compared to baseline. The advantage of utilizing general estimating equations was that it effectively increases the sample size (increasing power) and estimated more robust standard errors by taking into account the repeated measures and adjusting for covariates [32]. Generalized estimating equations can be used with non-normally distributed data and with sample sizes of 20 [33]. Further when missing data are random, all subjects can be retained in the analysis without imputation of missing data [34]. As dosage of the intervention was determined by the number of journal entries, it was a covariate in all of the analyses.

#### Specific aim #2

To determine the mechanisms of the Living with Hope Program, general estimating equation analysis was completed initially with Herth Hope Index scores as the dependent variable. The number of journal entries, General Self Efficacy Scale and Non Death Revised Grief Experience Inventory scores were entered into the model. In this way the factors that predicted hope were determined. This was then repeated with SF-12v2 (quality of life) summary scores as the dependent variable.

#### Specific aim #3

To describe what the caregivers perceive fosters their hope, the journal entries were transcribed and analyzed using Cortazzi’s [35] narrative analysis.

## Results

**Figure 3:**
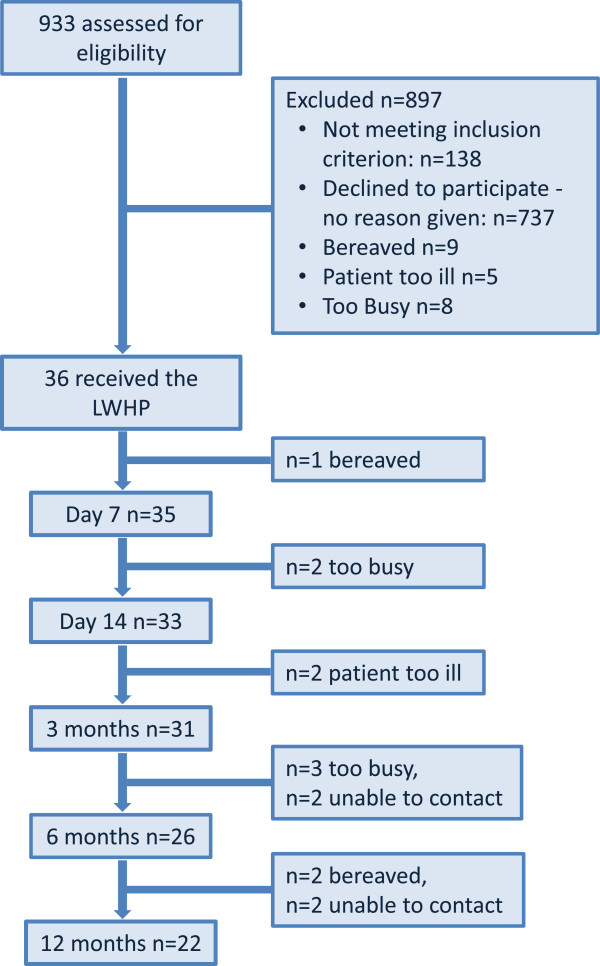
Sample Flow Diagram of Recruitment and Attrition.

Thirty six participants consented to participate. The number of participants at day 7 was 35; at day 14, 33; 3 months were 31; at 6 months was 26 and at completion of the study (12 months) was 22. Attrition throughout the study occurred due to factors not associated with the study, for example caregiver fatigue. Those who became bereaved during the intervention (days 7 and 14) were dropped from the study. Those who became bereaved during the remainder of the study were asked to continue. At 6 months seven participants were bereaved and at 12 months two were bereaved (see Figure 3 for a flow diagram of the sample).

The mean age was 59 (SD=11.6) and the majority were spouses [n=31(86.1%)]. The majority did not have any help with caregiving [n=18(50%)] and were not receiving any other services in addition to home care [n=21 (58.3%)]. The length of time they had been care giving was on average 32.41 months (SD=32.58). The majority of the family members they were caring for were male [n=34 (94.4%) male and n=2 (5.6%) female]. The care recipients were on average 65 years of age (SD 11=7.5%) and had a variety of cancer diagnoses. Table 1 presents additional demographic characteristics.

**Table 1:** Participant demographic variables: n=36

	**Frequency**	**Percent**
**Relationship to caregiver**		
Wife	31	86.1
Daughter	3	8.3
Common Law	2	5.6
**Marital status**		
Married	33	91.7
Divorced	2	5.6
Missing	1	2.8
**Ethnicity**		
Caucasian	33	91.7
First Nations	1	2.8
Asian	1	2.8
Missing	1	2.8
**Religious preference**		
Catholic	7	19.4
Protestant	20	55.6
Other	4	11.1
None	4	11.1
Missing	1	2.8
**Income**		
less than 10,000	3	8.3
10,000- 19,999	4	11.1
20,000- 29,999	3	8.3
30,000- 39,999	10	27.8
40,000- 49,999	2	5.6
50,000- 59,999	2	5.6
60,000 and more	9	25.0
Missing	3	8.3
**Patient medical diagnosis**		
lung	5	13.9
breast	1	2.8
prostate	3	8.3
colorectal	8	22.2
nasopharyngeal	3	8.3
lymphoma	3	8.3
urological	5	13.9
other	4	11.1
primary cancer not specified	4	11.1

All participants viewed the film and completed a mean of 4.18 (SD 4.07) journal entries per week with a total of 324 journal entries. They reported spending a mean of 9.12 minutes (SD= 8.89) per journal entry.

### Patterns of main variables over time

The mean, standard deviation and range of scores for the General Self Efficacy Scale, Non-Death Revised Grief Experience Inventory, Herth Hope Index and SF-12v2 Physical and Mental Health Summary at baseline, day 7, day 14, and 3, 6 and 12 months are presented in Table 2. Using general estimating equations Herth Hope Index scores at day 7 (β=1.83, p=0.048) and 12 months (β=2.71 p=0.013) were significantly higher than baseline values. General Self Efficacy Scale scores were significantly higher than baseline at all measured time points [day 7 (β=1.79, p=0.007), day 14 (β=1.44, p=0.035), 3 months (β=1.51, p=0.013), 6 months (β=1.90, p=0.002), 12 months (β=2.03 p=0.003)]. The Non-Death Revised Grief Experience Inventory scores were lower than baseline at four out of the five subsequent time points (day 7, day 14, and 6 and 12 months), but the changes were not statistically significant.

**Table 2:** GSES, NDRGEI, HHI and SF-12v2 at Day 7, 14, 3, 6, 9 and 12 months

**Variable**	**Baseline**	**Day 7**	**Day 14**	**3 months**	**6 months**	**12 months**
	**N=36**	**N=35**	**N=33**	**N=31**	**N=26**	**N=22**
GSES	31.1(3.94)	33.04(4.03)*	32.44(4.41)*	32.63(3.64)*	33.2(3.74)*	33.21(4.44)*
NDRGEI	72.82(23.92)	71.75(24.13)	70.22(22.23)	73.26(23.6)	64.63(25.59)	66.29(24.43)
HHI	37.79(5.97)	39.74(4.96)*	39.06(6.05)	38.17(5.22)	39.34(4.96)	40.53(5.2)*
SF12 Physical health	45.17(3.87)	44.36(4.47)	45.56(5.05)	45.10(5.31)	43.98(4.67)	43.37(5.28)*
SF12 Mental health	43.59(5.35)	45.27(5.66)	44.15(6.40)	45.57(5.76)*	44.52(5.66)	47.15(5.36)*

*P< 0.05.

The SF-12v2 physical summary score at 12 months (β=−1.83, p=0.04) was significantly lower than the baseline value. Scores at other data time points were not statistically significant. The SF-12v2 mental health summary scores at 3 months (β =1.87, p=0.03) and 12 months (β=3.34, p=0.003) were significantly higher than baseline scores.

In comparing the means of the SF-12v2 data to United States population norms, over all time points, the physical health summary scores were below the 25th percentile (46.53) and just above the 25th percentile (45.13) for the mental health summary scores. Over all study time points the SF-12v2 physical and mental health scores were below the general population norm (mean of 50 found in the 1998 General US population norms).

There were no other significant changes over time. Demographic variables were not significantly associated with changes in Herth Hope Index, General Self Efficacy Scale, and Non-Death Revised Grief Experience Inventory and SF12-v2 scores.

### Mechanisms of the living with hope program (testing of the model)

With the Herth Hope Index scores as the dependent variable, General Self Efficacy Scale (p<0.001) and Non-Death Revised Grief Experience Inventory (p=0.033) scores were significant (Table 3). As General Self Efficacy Scale scores increased, so did the Herth Hope Index scores, showing positive correlations. As the Non-Death Revised Grief Experience Inventory scores decreased, Herth Hope Index scores increased, as they were negatively correlated. General Self Efficacy Scale and Non-Death Revised Grief Experience Inventory scores were predictors for changes in the Herth Hope Index scores at day 7 and 12 months.

**Table 3:** Model of hope as dependent variable and GSES and NDGREI

**Parameter**	**B**	**Std. error**	**95% Confidence interval**		**p-value**
			**Lower**	**Upper**	
Time points (Baseline)					
12 Months	2.89	1.21	0.52	5.26	0.02
6 Months	−1.51	1.78	−5.00	1.97	0.40
3 Months	−1.94	1.19	−4.26	0.39	0.10
Day 14	0.80	1.09	−1.34	2.93	0.46
Day 7	1.19	0.87	−0.51	2.89	0.17
Number of journal entries	−0.06	0.09	−0.24	0.11	0.46
Self-Efficacy	0.67	0.13	0.41	0.93	<0.001*
Grief and loss	−0.05	0.03	−0.11	0.00	0.033*

*p<0.05.

**Figure 4:**
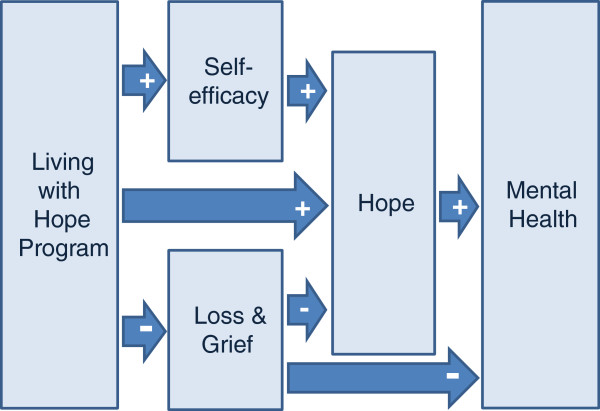
Revised Living with Hope Conceptual Model.

With SF12 v2 physical and mental health summary scores as dependent variables, Herth Hope Index scores (p<0.001) and Non-Death Revised Grief Experience Inventory scores (p=0.01) were found to be significant predictors for Mental Health Summary scores (Table 4). They were also significant predictors for Physical Health Summary Scores (Herth Hope Index p=0.01; Non-Death Revised Grief Experience Inventory p= 0.04). For SF-12v2 mental health summary scores, as the Herth Hope Index scores increased and the Non-Death Revised Grief Experience Inventory scores decreased, SF-12 v2 mental health summary scores increased. The revised model based on the findings for mental health summary score is shown in Figure 4. However for the SF-12v2 physical health summary scores (see Table 5) as the Herth Hope Index scores increased and Non-Death Revised Grief Experience Inventory scores decreased-physical health decreased. In order to determine if there were variable interaction affects occurring, variables that were entered into the multivariate analysis with SF-12v2 physical health summary scores were removed one by one to determine if there were changes in the direction of the relationships. None were noted.

**Table 4:** Model of mental health summary score as dependent variable

**Parameter**	**B**	**Std. error**	**95% Confidence interval**		**p-value**
			**Lower**	**Upper**	
Time points (Baseline)					
12 Months	1.03	4.81	−8.41	10.46	0.83
6 Months	6.95	6.51	−5.81	19.71	0.29
3 Months	0.49	4.07	−7.49	8.47	0.90
Day 14	0.70	3.27	−5.71	7.10	0.83
Day 7	−2.09	3.03	−8.03	3.85	0.49
Number of journal entries	−0.09	0.31	−0.69	0.51	0.77
Self-Efficacy	0.21	0.39	−0.55	0.97	0.59
Grief and loss	−0.26	0.10	−0.45	−0.06	0.01*
Hope Herth index	1.16	0.35	0.47	1.84	<0.001*

*p<0.05.

**Table 5:** Model of physical health summary score

**Parameter**	**B**	**Std. error**	**95% Confidence interval**		**p-value**
			**Lower**	**Upper**	
Time points (Baseline)					
12 Months	−5.26	4.66	−14.40	3.89	0.26
6 Months	−0.01	2.22	−4.36	4.35	0.99
3 Months	0.48	1.19	−1.86	2.82	0.69
Day 14	1.23	0.96	−0.66	3.12	0.20
Day 7	0.02	0.62	−1.19	1.22	0.97
Number of journal entries	−0.03	0.08	−0.18	0.13	0.74
Self-Efficacy	−0.21	0.14	−0.49	0.06	0.13
Grief and loss	0.04	0.02	0.00	0.07	0.04*
Hope Herth index	−0.20	0.08	−0.35	−0.05	0.01*

*P<0.05.

### Descriptions of what influences hope

The data from the qualitative analysis supported the quantitative data results suggesting that the Living with Hope Program increased participants’ hope. several participants described how the *Living with Hope Program,* in particular writing each day in “Stories of the Present” helped them to maintain hope: For example one participant wrote: *“My hope is seeing the positive and also the exploring and facing my fears for a defined period each day… journaling was a time to honestly address my fears, and to become a better person.”* Another participant wrote *“Hope [came] from within and the gift of reflection [journaling], hope [came] from faith, family and friends.”*

Other influences on the participants’ hope included the specific circumstances of each day, social support and faith and spirituality, Specific circumstances such as accessing health care was a theme found in all the journals the participants lived in rural areas, travel to obtain health care added to their stress and decreased their hope. For example one participant described her day: “*have to spend the whole day driving 6 hours & waiting 3 for chemo & then a doctor’s appointment after that. It’s a hard job & it’s hard to stay calm till we’re all done*”. Financial stress was evident as well: *“have been paying bills – bills – bills – it is very hard to be hopeful”* As well the caregivers’ level of hope was influenced by the mood of the care recipient and the care receiver’s state of health. For example one participate wrote: *“He is confused and it hurts so much to hear him!”*

*Social support* was described as fostering hope, whether this was support from family members, friends or health professionals such as doctors and nurses. For example as one participant wrote: “*We have such wonderful friends and family. They bring supper almost every day*.” This support, brought hope to their day and to their lives. Some participants also found hope through their *faith and spirituality.* The belief in something bigger than them was experienced as supportive. For example, one participant wrote: *“I know God is in charge & we have to trust him, his ways are not always the way we want them to be”.* The findings of the full narrative analysis of the qualitative data was submitted for publication in a separate manuscript.

## Discussion

The study findings suggest that the Living with Hope Program shows promise in increasing hope in rural women caregivers of persons with advanced cancer after one week compared to baseline scores.

Several hope focused interventions have been found to be effective in fostering hope in other populations such as persons with advanced cancer [16], recurrent cancer [36] and newly diagnosed cancer patients [37]. A recent review of intervention studies for caregivers of persons with cancer, however, did not identify any psychosocial hope focused interventions [38]. Thus the Living with Hope Program is unique and may address this gap in knowledge. Changes in the hope score occurred at day 7 and 12 months. Although the sample size was small, there is the possibility that the Living with Hope Program has a short effect and does not have an impact over time. In Herth’s [17] evaluation of a hope intervention for persons with recurrent cancer, there were significant positive changes in hope and quality of life over time (3, 6 and 12 months). Herth’s intervention consisted of eight two hour hope focused interventions with a skilled health care professional over an eight week time period. This type of intervention would not be feasible for rural caregivers actively caring for persons with advanced cancer at home. Herth’s results, however, suggested that a hope intervention may have longitudinal effects. More research is needed with larger sample sizes and possibly viewing the film more than once and extending the journaling exercise of the Living with Hope Program over time.

The testing of the model suggests that the possible mechanism by which the Living with Hope Program increases hope was through increasing feelings of self –efficacy (confidence in the ability to deal with difficult situations) and decreased feelings of loss and grief. The model also suggested that hope predicted mental health summary scores. This hypothesis was supported in the data. Loss and grief were also predictors of mental health summary scores. The qualitative data from the journals supported this finding, with participants, suggesting that the Living with Hope Program helped them to address their fears and find the positive in their situation. The model representing the mechanisms through which the Living with Hope Program was effective was revised based on these findings.

The model did not include demographic variables and physical health as there were no statistically significant associations found among the demographic variables with the main variables and no significant changes over time in participants’ physical health summary scores. Of concern in this study is the negative relationship of general self-efficacy and hope with physical health summary scores and the positive loss and grief relationship. Two other studies have reported unexplainable relationships with the SF-12 physical health summary scores and other psychological measures [39,40]. These authors suggest that SF-12 physical health summary scores does not correlate with psychological measures. As a result, these results were not added to the revised model. Future studies should use more valid and reliable quality of life measures.

The physical and mental health summary scores clearly indicate the poor physical and mental health of the participants. Although research studies have established the impact of family caregiving on caregivers and rural Canadians have reported poorer health status than their urban counterparts [41], this is the first study to compare their health to population norms. Physical and mental health scores using the SF-12v2 compared to normative population scores in the United States, suggest that the participants’ physical and mental health were well below population norms (at the 25 percentile or less). These findings underscore the need to monitor the effects of caregiving on rural caregivers’ physical and mental health and for practical support of rural women caregivers of persons with advanced cancer. Fostering their inner resource of hope is only one mechanism to achieve that goal.

### Limitations

There are several limitations to this study that include study design and sample characteristics. A quasi-experimental time series design compares changes, not to a control group, but rather to the participants themselves. As well, it does not involve randomization. The design was chosen based on its suitability to the study purpose and the nature of the population. Harding and Higginson [42], in a systematic review of interventions in palliative care suggested that interventions should be evaluated using repeated measures from baseline and that ideal randomized controlled trials may be inappropriate. These design recommendations were supported by Grande and Todd [43] following their review of randomized control trials in palliative care research. Grande and Todd also recommended using mixed method designs (quantitative and qualitative) to improve interpretation of the results.

The small sample size reflected the difficulties in accessing and recruiting potential participants. The findings specific to the low physical and mental health scores of the participants, provide insight as to why recruitment was difficult. In a qualitative study of rural caregivers of family members with advanced disease, the participants described the multiple significant transitions they experienced in caring for their family member [3]. These included significant changes in their own physical and mental health. It is difficult then for rural women caregivers, who were dealing with their own health issues as well as trying to provide the care to their family member with advanced cancer, to take on the burden of participating in a research study. The small sample size does limit the generalizability of the findings. However, in spite of the small sample size, there were significant study results, suggesting that the Living with Hope Program shows promise in increasing feelings of self-efficacy, decreasing loss and grief and increasing hope in this high-risk population.

## Conclusions

The Living with Hope Program for family caregivers of persons with advanced cancer is a promising, practical psychosocial supportive hope program that may foster hope. Hope is a psychological inner resource that helps caregivers deal with the caregiving experience. Family care giving is what sustains patients at the end of life [44] and with changing demographics and diminishing resources there is a potential that every Canadian will be an informal caregiver at some time [45]. The Living with Hope Program offers a unique and innovative approach that has the potential to be one strategy to support family caregivers in this difficult journey.

## Competing interests

The authors declare that they have no competing interests.

## Authors’ contributions

WD, AW, RT, DC, LH, LH and MH conceptualized the study and obtained funding. WD as nominated PI was responsible for the overall study coordination including recruitment, data collection, transcription of the data and wrote the initial draft of the manuscript. SG was responsible for the statistical analysis. All authors contributed to the manuscript by submitting comments and suggestions. All authors read and approved the final manuscript.

## Pre-publication history

The pre-publication history for this paper can be accessed here:

http://www.biomedcentral.com/1472-684X/12/36/prepub
